# Supplementary material for: Causal Relationship between Aging and Anorexia Nervosa: A White-Matter-Microstructure-Mediated Mendelian Randomization Analysis
Source: Biomedicines. 2024 Aug 16;12(8):1874. doi: 10.3390/biomedicines12081874 (PMC11351342; doi:10.3390/biomedicines12081874)
Supplement: Supplementary file 1 [file biomedicines-12-01874-s001.zip › Supplementary Figure/Figure S4/Singe SNP plot of MR analysis for TL to ubm-b-1639.pdf]

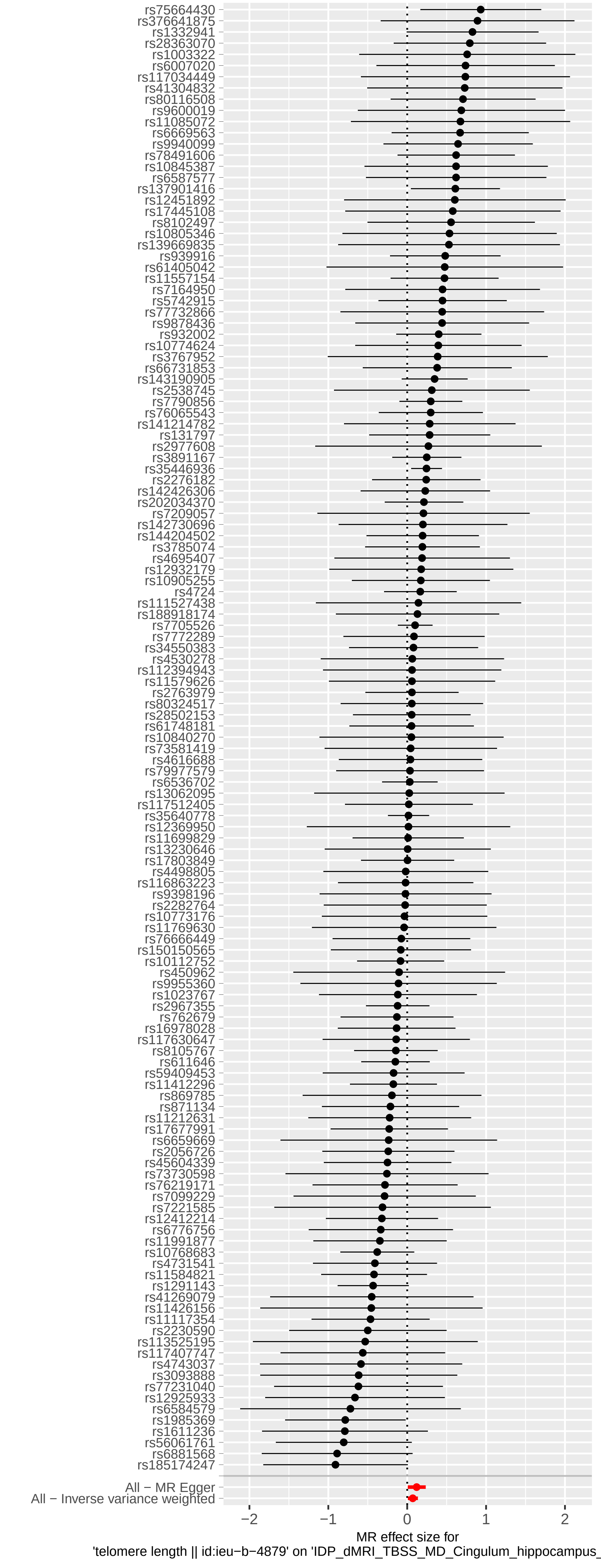
rs75664430
rs376641875
rs1332941
rs28363070
rs1003322
rs6007020
rs117034449
rs41304832
rs80116508
rs9600019
rs11085072
rs6669563
rs9940099
rs78491606
rs10845387
rs6587577
rs137901416
rs12451892
rs17445108
rs8102497
rs10805346
rs139669835
rs939916
rs61405042
rs11557154
rs7164950
rs5742915
rs77732866
rs9878436
rs932002
rs10774624
rs3767952
rs66731853
rs143190905
rs2538745
rs7790856
rs76065543
rs141214782
rs131797
rs2977608
rs3891167
rs35446936
rs2276182
rs142426306
rs202034370
rs7209057
rs142730696
rs144204502
rs3785074
rs4695407
rs12932179
rs10905255
rs4724
rs111527438
rs188918174
rs7705526
rs7772289
rs34550383
rs4530278
rs112394943
rs11579626
rs2763979
rs80324517
rs28502153
rs61748181
rs10840270
rs73581419
rs4616688
rs79977579
rs6536702
rs13062095
rs117512405
rs35640778
rs12369950
rs11699829
rs13230646
rs17803849
rs4498805
rs116863223
rs9398196
rs2282764
rs10773176
rs11769630
rs76666449
rs150150565
rs10112752
rs450962
rs9955360
rs1023767
rs2967355
rs762679
rs16978028
rs117630647
rs8105767
rs611646
rs59409453
rs11412296
rs869785
rs871134
rs11212631
rs17677991
rs6659669
rs2056726
rs45604339
rs73730598
rs76219171
rs7099229
rs7221585
rs12412214
rs6776756
rs11991877
rs10768683
rs4731541
rs11584821
rs1291143
rs41269079
rs11426156
rs11117354
rs2230590
rs113525195
rs117407747
rs4743037
rs3093888
rs77231040
rs12925933
rs6584579
rs1985369
rs1611236
rs56061761
rs6881568
rs185174247
All − MR Egger
All − Inverse variance weighted
−2
−1
0
1
2
MR effect size for
'telomere length || id:ieu−b−4879' on 'IDP_dMRI_TBSS_MD_Cingulum_hippocampus_
